# A Necropsy Study of Disease and Comorbidity Trends in Morbidity and Mortality in the Koala (*Phascolarctos cinereus*) in South-East Queensland, Australia

**DOI:** 10.1038/s41598-019-53970-0

**Published:** 2019-11-25

**Authors:** V. Gonzalez-Astudillo, J. Henning, L. Valenza, L. Knott, A. McKinnon, R. Larkin, R. Allavena

**Affiliations:** 1 School of Veterinary Science, The University of Queensland, Building, 8114 Gatton, Queensland Australia; 2 Moggill Koala Rehabilitation Centre, Department of Environment and Science, 55 Priors Pocket Rd, Moggill, Queensland 4070 Australia; 3 Ipswich Koala Protection Society, Mt Forbes, Queensland 4340 Australia; 4 Present Address: California Animal Health and Food Safety Laboratory, UC Davis, 105 W Central Ave, San Bernardino, CA 92408 USA

**Keywords:** Biodiversity, Diseases

## Abstract

Koalas are an iconic Australian marsupial undergoing precipitous population reduction in South-East Queensland from complex interacting threats. To investigate the causes of death and the interaction of comorbidities with demography in South-East Queensland koalas, a large scale, high-throughput prospective necropsy survey was conducted spanning 2013–2016. During this period, 519 necropsies were conducted in 155 young/subadult koalas, 235 mature, 119 old koalas and 10 of unknown age. Similar numbers of males and females were assessed. Trauma and infectious disease at were the most common single diagnoses. However, comorbidity was frequent, including multicentric infection or infectious disease in combination with trauma or senescence. Female koalas had proportionally more reproductive chlamydiosis compared to males in which the ocular and urinary systems were more commonly affected. Comorbidity and disease were strongly associated with poor body condition, and trauma was associated with good body condition. Animals affected by motor vehicle trauma were often in better body condition than those affected by animal attack, tree fall or other causes of trauma. This study identified a higher frequency of infections and comorbidity then previously reported, confirming the complex nature of interacting threats to the koala population.

## Introduction

The koala (*Phascolarctos cinereus*) is a medium-sized, arboreal folivorous marsupial, with a broad but fragmented distribution associated with *Eucalyptus* spp. woodlands, its primary food source^1^. The species is listed nationally as vulnerable in response to population declines in the states of Queensland (QLD) and New South Wales (NSW), and the Australian Capital Territory^2,3^. Despite being overly abundant in the southern extension of their range, koalas in South-East Queensland (SEQLD) are threatened by the population limiting effects of disease^4,5^ and habitat clearing for urbanization^6^, which exposes koalas to trauma from vehicle collisions^7^ and animal attacks^8^. Despite investments in medical care and species management, there has been a rapid population decline from 1996 through to 2014, with declines of 80% in the Koala Coast and 54% in Pine Rivers in surveyed Queensland populations^9,10^.

A recent retrospective epidemiological study using passive surveillance hospital records of koala mortality spanning 17 years determined several major factors drive koala hospital submissions. Vehicle collisions, and chlamydiosis-associated debilitation and infertility were the major causes of mortality and morbidity^10,11^. However, this study was based on retrospective medical records and necropsies were not conducted in all animals, impeding the detailed interrogation of comorbidity and disease interaction^10^. Given the complex threats affecting the koala population and the high prevalence of disease and injury found in this retrospective study, a passive surveillance necropsy study was undertaken.

Passive surveillance utilizes medical records or data produced by other health-related activities^12^, and is an increasingly popular method for wildlife studies due to its cost-effectiveness and the feasibility of collecting information across multiple seasons. In this study, a passive surveillance method was used to recruit koalas for detailed necropsy examination. The purpose of this prospective pathology study was to apply systematic necropsy and data recording methodology to accurately identify causes of mortality and to interrogate the interplay of comorbidities driving terminal koala submissions to hospitals in SEQLD. This is the one of the most extensive pathological studies applied to a declining wild species in Australia, identifying major causes of death, comorbidity trends and permitting the statistical evaluation of variables influencing threats to the species.

## Results

### Diagnoses of koalas admitted

Necropsies were conducted on 519 koalas. The male-to-female ratio was 1.064 (males: 51.4%, N = 266; females: 48.4%, N = 250; Hermaphrodite: 0.2%, N = 1; sex not recorded in N = 2). The age of koalas differed significantly between sexes (p < 0.001) with more older females compared to males being submitted [old: 61.9% (N = 73) females versus 38.1% (N = 45) males; mature: 45.3% (N = 106) females versus 54.7% (N = 128) males; young: 45.5% (N = 67) females versus 54.5% (N = 58) males; unknown: 40% (N = 4) females and 60% (N = 6) of males].

A total of 29 different diagnoses were made (Supplementary Table 1). A total of 299/519 koalas (57.6%, 95%CI: 53.2–61.9%) were found with co-occurring conditions (referred to as co-morbidities), while 220/519 koalas (43.3%, 95%CI: 38.1–46.8%) were diagnosed with a single condition. The single most common diagnosis in koalas was trauma (42.7%, Table 1) followed by infectious disease (31.8%), while the highest co-morbidity was multicentric infections (32.1%), followed by combinations of infectious disease and trauma (18.4%). Across the 29 diagnoses made on all 519 koalas necropsied, infectious processes were detected in 63.4% (329/519) of cases and trauma in 36.4% (189/519) of cases.

**Table 1:** Percentage of diagnoses (N of diagnoses) of the four most common single (N = 220 koalas) and co-occurring (N = 299 koalas) diagnoses made on koalas submitted to wildlife hospitals and receiving necropsies at The University of Queensland between between April 2013 and July 2016 in South-East Queensland, Australia.

Type of diagnoses	Single diagnosis	Co-occurring diagnoses
Infectious	31.8% (70)	
Infectious (multicentric)		32.1% (96)
Infectious/other diseases		7.7% (23)
Infectious/senescence		15.1% (45)
Infectious/trauma		18.4% (55)
Trauma	42.7% (94)	
Wasting unknown cause	7.7% (17)	
Neoplasia	6.4% (14)	

Co-morbidities differed significantly (p < 0.001) between male and female koalas, with 69.6% (174/250; 95%CI: 63.5–75.2%) of females having co-morbidities, but only 46.6% (124/266; 95%CI: 40.5–52.8%) of males. Not surprisingly, co-morbidities differed between age groups, being most common among old koalas (98/118; 83.1%, 95%CI: 75.0–89.3%), followed by mature koalas (123/234; 52.6%, 95%CI: 45.9–51.1%) while still 46.7% of young koalas (72/154; 95%CI: 38.7–54.9%) were diagnosed with more than one disease or health problem. Interestingly, among the mature and young koalas, a larger proportion of females had co-morbidities (p = 0.002 and p = 0.006, respectively), while co-morbidities did not differ between sexes for old koalas (p = 0.128).

Across the 516 koalas that were classified as males or females, there was a significantly larger proportion of females (187/250; 74.8%) affected by infectious diseases (alone or in combination with other conditions) compared to males (141/266; 53.0%) (p < 0.001). However, trauma and poor body condition, termed wasting, were more common in males compared to female animals with 41.4% of males (110/266) affected by trauma (alone or in combination with other conditions) compared to 31.6% females (79/250) (p = 0.023) and 10.5% of males (28/266) suffering from wasting from unknown causes (alone or in combination with other conditions) compared to 1.2% females (3/250) (p < 0.001). No sex differences were observed for neoplasia (females: 20/250; 8%; males: 21/266; 7.9%; p = 1.0)

We then explored for each age group associations between sex and the occurrence of each of the most important diagnoses (infectious, trauma, neoplasia and wasting of unknown cause) occurring alone or in combination with other conditions versus all other diagnoses. While there was no statistical association (p < 0.05) between sex and neoplasia for each of the three age groups, a larger proportion of females across all age groups (compared to males) were diagnosed with infections (young: p < 0.001, mature: p = 0.017, old: p = 0.019) (Table 2). More old males were diagnosed with trauma compared to old females (p = 0.018), but this gender difference was not present for young and mature animals (Table 2). Notably, young and mature males were more likely to have wasting of unknown cause than females (p < 0.001 and p = 0.041, respectively). Wasting of unknown cause did not occur in old animals as poor body condition in this class was assumed to be caused by dental attrition.

**Table 2:** Percentage of koalas (N of koalas) of the four most common diagnoses by sex (stratified by age class; N = 509 koalas with age and sex information) for koalas submitted to South-East Queensland hospitals and receiving necropsies at The University of Queensland from 2013 through to 2016. Koalas may be counted more than once in the diagnoses categories due to co-morbidities.

Age group	Infectious		Trauma		Neoplasia		Wasting unknown	
	Female	Male	Female	Male	Female	Male	Female	Male
Young/Subadult (N = 154)	67.2% (45/67)*	35.6% (31/87)*	46.3% (31/67)	55.2% (48/87)	4.5% (3/67)	5.8% (5/87)	1.5% (1/67)*	60.7% (17/87)*
Mature (N = 234)	76.4% (81/106)*	61.7% (79/128)*	32.1% (34/106)	32.8% (42/128)	9.4% (10/106)	9.4% (12/128)	1.9% (2/106)*	39.3% (11/128)*
Old (N = 118)	80.8% (59/73)*	60.0% (27/45)*	17.8% (13/73)*	37.8% (17/45)*	8.2% (6/73)	6.7% (3/45)		

^*^Statistical differences between sex groups at p < 0.05.

Percentage of koalas per sex group within each age class for each diagnosis was calculated as N of koalas per sex and age group for each diagnoses/Total number of koalas per sex and age group.

**Figure 1:**
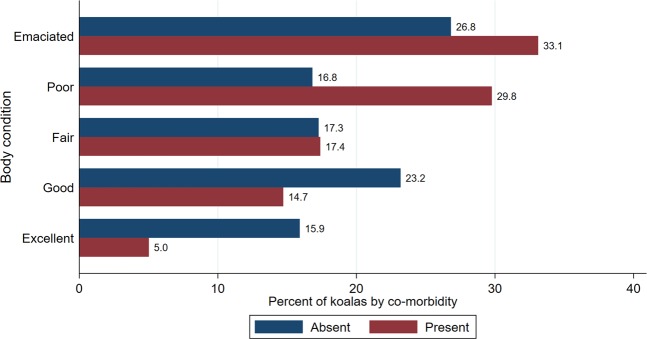
Percentage of koalas by body condition category with co-morbidities absent or present (N = 519). Koalas were submitted to South-East Queensland hospitals and receiving necropsies at The University of Queensland from 2013 through to 2016.

Notably, 30.4% (158/519) of koalas were submitted in emaciated body condition, followed by 24.3% (126/519) koalas in poor, 17.3% (90/519) in fair, 18.3% (95/519) in good and only 9.6% (50/519) in excellent body condition. We also explored the association between body condition and co-morbidities (Fig. 1). Koalas with co-morbidities were more likely to be in detrimental body condition compared to koalas with a single condition (p = 0.001).

### Infectious disease

**Figure 2:**
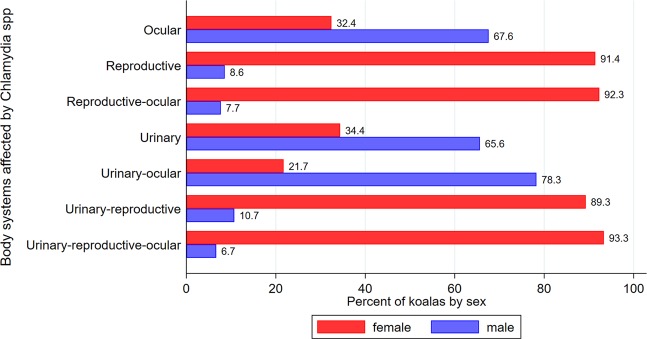
Percentage of koalas by sex with gross lesions compatible with chlamydiosis across body systems (N = 304 koalas with *Clamydia*spp. infection). Koalas were submitted to South-East Queensland hospitals and receiving necropsies at The University of Queensland from 2013 through to 2016.

Of the total of 63.4% (329/519) cases with infectious processes (alone or in combination with other conditions), 92.4% (304/329) had hallmark lesions of *Chlamydia* spp. infection. The percentage of koalas by sex for each body system (alone or in combination with other body systems) affected by *Chlamydia* spp. are shown in Fig. 2. There were significant differences between female and male koalas in the body system affected (p < 0.001), with the reproductive system alone or in combination with other body systems affecting about 90.0% of female koalas, while ocular, urinary or urinary-ocular infections were observed between 65–78% of male koalas. The distribution of body systems affected by chlamydiosis for each sex strata are shown in Supplementary Fig. 1, highlighting that the urinary tract was the most common body system affected in males (52.5% of all Chlamydiosis cases in males) and the urinary-reproductive tracts the most frequent body system affected in females (41.0% of all Chlamydiosis cases in females). Urogenital lesions were diagnosed in 87.8% (267/304) of *Chlamydia* spp. infections, frequently manifesting as cystitis (65.9%; 176/267). Two representative male koalas with classical urogenital chlamydiosis pathology were tested and were verified positive to *C. pecorum* via real-time PCR.

### Urinary disease

**Figure 3:**
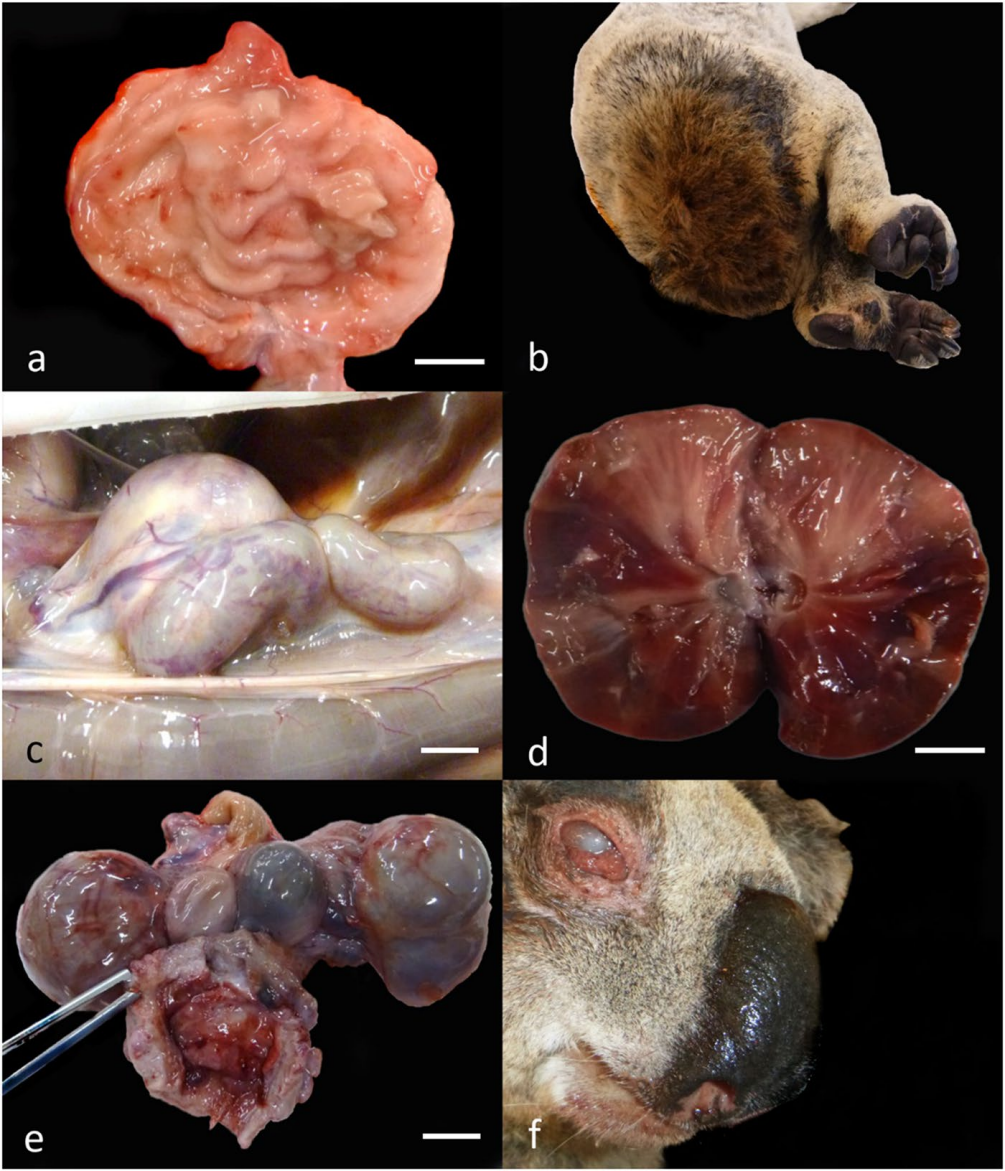
Gross lesions associated with chlamydiosis in koalas. (**a**) Urinary bladder with chronic cystitis. (**b**) ‘Wet bottom’ secondary to chronic cystitis. (**c**) Hydroureter associated with ascending urinary tract infection. (**d**) Chronic nephritis. (**e**) Bilateral bursitis and haemorrhagic cystitis. (**e**) Chronic conjunctivitis. All bars = 1 cm. Figures (**a–d**) extracted and modified from Gonzalez-Astudillo, 2019^10^.

Cystitis was characterized grossly by thickening and firmness of the urinary bladder wall, with mucosal infolding and occasional petechiae (Fig. 3a). Soiling around the common vestibule colloquially referred to as ‘wet bottom’^10,13^, was common in chronic cases (Fig. 3b). Clinical chlamydial scoring was recovered from medical records for 43 of the 176 cystitis cases, with 58.1% (25/43) severe and 18.6% (8/43) moderate grade. The predominant histological lesion was moderate-to-severe fibrosis accompanied by detrusor muscle hypertrophy, transmural lymphoplasmacytic infiltrates, and occasional urothelial ulceration. Chronic cystitis resulted in obstruction of the bladder outlet due fibrosis and myohypertrophy in the bladder wall.

Renal complications compatible with chlamydial infection affected 34.8% (106/304) of all koalas and included 9/106 cases of hydronephrosis (Fig. 3c) and 13/106 of renal pyramidal fibrosis from chronic chlamydial pyelonephritis (Fig. 3d). Primary renal disease was also observed without cystitis in 51.8% (43/83) cases. The predominant histological lesion was lymphocytic interstitial nephritis or chronic lymphocytic segmental pyelonephritis. Severe renal disease was infrequent, and included ascending urinary tract infection - UTI (4 cases), end stage kidney disease (4 cases), and pyelonephritis (10 cases). Renal crystal precipitation was observed in 21 cases, mostly morphologically consistent with struvite in 47.6% (10/21) cases, and calcium oxalate in 23.8% cases (5/21).

### Reproductive disease

Reproductive lesions consistent with chlamydia infection, predominantly cystic dilation of the ovarian bursa (bursitis) (Fig. 3e), occurred in 66.6% (122/183) female cases with *Chlamydia* spp. infections at necropsy. In 3.8% (7/183) of these cases, chronic bursitis was found along with gross evidence of salpingitis (acute or chronic). Association of bursitis with fibrotic salpingitis was observed but not consistently recorded. Fluid within the bursa varied from clear and straw-colored, to purulent or hemorrhagic. The bursa was bilaterally compromised in 55.7% (68/122) cases. The main histological feature was replacement of ovarian and fallopian structures with mature collagen, occasionally with lymphocytic infiltration. Infection of other reproductive tract sites co-occurred with bursitis in 22.1% (27/122) of cases, primarily fibrosis or suppurative infection of the uteri. In the male, 64.2% (9/14) of cases of urogenital infections were consistent with chlamydiosis, with cystitis and urethritis most common. Ten of fourteen cases had chronic multifocal prostatitis with intraglandular pustules.

### Ocular disease

Ocular lesions consistent with chlamydiosis were diagnosed in 28.9% of koalas (88/304 *Chlamydia* spp. infections) (Fig. 3f). Chlamydial clinical scoring was recovered for 46.6% (41/88) of cases and frequently was high-grade (85.3%; 35/41; score ≥2). Ocular chlamydiosis was macroscopically a chronic, bilateral (60/88; 68.1%) mucopurulent conjunctivitis/keratoconjunctivitis, with periocular fur matting or alopecia, and palpebral adhesion.

A total of 87.5% (77/88) of ocular disease cases occurred with multisystemic chlamydiosis, and occasionally ‘other diseases’ or dental attrition. Koalas solely with ocular disease rarely had low BC 9.1% (8/88). Animals with ocular disease and low BC typically had a combination of multisystemic comorbidities (67.0%; 59/88).

### Trauma

Of the 36.4% (189/519) koalas diagnosed with trauma (alone or in combination with other conditions), based on hospital records, 57.7% (109/189) cases were from motor vehicle collisions, 21.2% (40/189) were from animal attacks (dogs, livestock), 6.9% (13/189) cases corresponded to tree falls, and in 14.3% (27/189) the cause was unknown (Table 3). Acute bone fractures affected 46.0% (87/189), mostly located in the head in 55.1% cases (104/189) or in long bones in 34.5% cases (65/189). Severe hemoabdomen and hemothorax from hepatic and pulmonary lacerations were frequent. Healed bone fractures were infrequent but two cases of proliferative osteophytic periosteal masses compatible with fracture calluses were observed. A total of 10/19 females with evidence or history of carrying pouch or back young perished to trauma caused by motor vehicles.

**Table 3:** Percentage of koalas (N of koalas) by trauma type with and without co-morbidities  (N = 189 koalas with trauma) for koalas submitted to South-East Queensland hospitals and receiving necropsies at The University of Queensland from 2013 through to 2016.

Type of trauma	Percentage koalas (N koalas)
***Vehicle collisions***	
Healthy	56.9% (62)
All other diagnosis/trauma	39.5% (43)
Wasting unknown cause/trauma	3.7% (4)
*Total*	100% (109)
***Animal attacks***	
Healthy	42.5% (17)
All other diagnosis/trauma	47.5% (19)
Wasting unknown cause/trauma	10.0% (4)
*Total*	100% (40)
***Tree falls***	
Healthy	23.1% (3)
All other diagnosis/trauma	46.1% (6)
Wasting unknown cause/trauma	30.8% (4)
*Total*	100% (13)
Other causes^*^	
Healthy	44.4% (12)
All other diagnosis/trauma	48.2% (13)
Wasting unknown cause/trauma	7.4% (2)
*Total*	100% (27)

^*^Trauma by other causes includes categories such as intraspecific trauma, heat stress, and undetermined cause.

**Figure 4:**
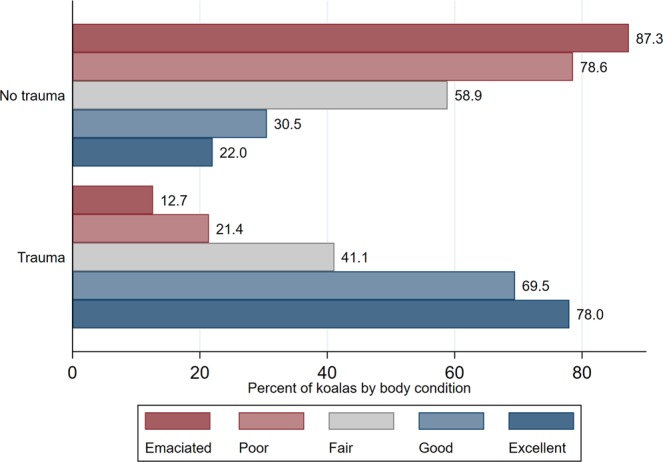
Percentage of koalas with and without trauma by body condition category (N = 519). Koalas were submitted to South-East Queensland hospitals and receiving necropsies at The University of Queensland from 2013 through to 2016.

In general, koalas with trauma (alone or in combination with other conditions) had better body condition than koalas submitted for other causes than trauma (p < 0.001) (Fig. 4).

**Figure 5:**
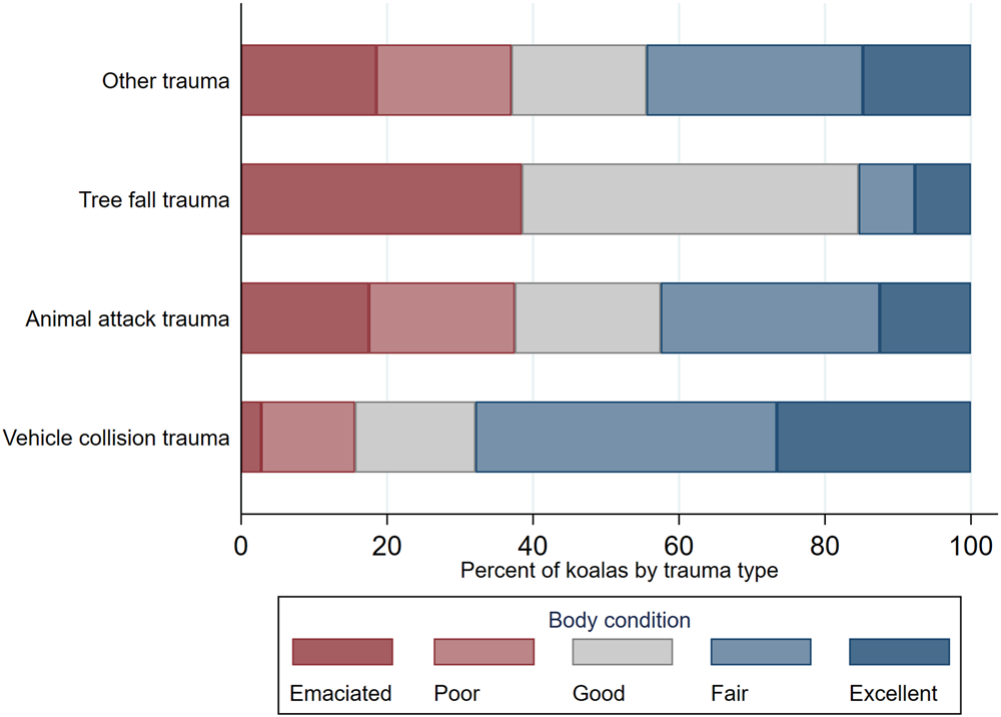
Percentage of koalas by trauma type and body condition category (N = 189 koalas with trauma). Koalas were submitted to South-East Queensland hospitals and receiving necropsies at The University of Queensland from 2013 through to 2016.

We further explored the relationship between body condition and type of trauma. Koalas with trauma due vehicle collision had significantly better body condition than koalas being affected by animal attack, tree fall, and other trauma causes (p < 0.05) (Fig. 5).

### Neoplastic disease

Neoplasms were detected in 7.9% (41/519) koalas alone or in combination with other conditions. Round cell tumors comprised 58.0% (24/41), including lymphomas (20 cases) and leukemias. Mesenchymal neoplasms affected 39.0% (16/41) of koalas, including cranial^14^ and costal osteochondromas (7 cases) and primary serosal myxosarcoma^15^ (2 cases). The least common neoplasms (15%) were epithelial, including colonic and hepatic adenocarcinomas, squamous cell carcinoma, and a bronchioloalveolar carcinoma. Thirty-nine percent (16/41) neoplasms co-occurred with chlamydiosis, but only 5.0% (2/41) co-occurred with trauma.

### Miscellaneous findings

Pulmonary lesions were found in 4.0% (21/519) of koalas including suppurative pneumonias and bronchopneumonias. Focal or interstitial fibrosis indicating chronicity was found in 33.3% (7/21) of koalas with respiratory lesions. Cases with rare or novel pathogens included a *Psynchrobacter* spp. pleuritis, a pulmonary abscess caused by *Mycobacterium abscessus*, and a pulmonary phaeohyphomycosis caused by *Cladosporium* spp., all PCR-confirmed.

**Figure 6:**
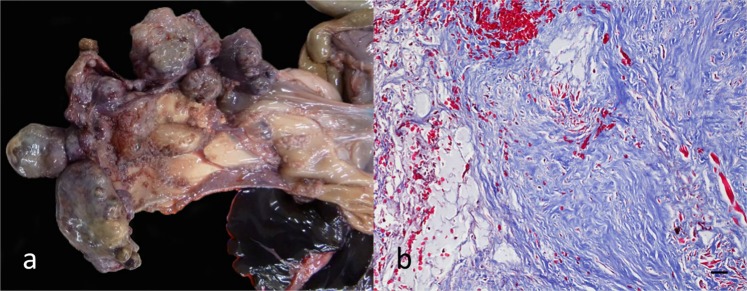
Koala fibromatosis syndrome. (**a**) Fibrotic nodules efface the splenic parenchyma. (**b**) Histopathology of fibromatosis lesion. Masson’s trichrome stain, 20x magnification. Bar = 30 µm. Figures in this panel extracted from Gonzalez-Astudillo, 2019^10^.

Seven koalas of the 519 koalas presented with multinodular masses in the spleen (Fig. 6a), termed by the authors “koala fibromatosis syndrome”. Nodules were formed by well-differentiated fibrocytes, which effaced and infiltrated the spleen (Fig. 6b) and liver (3 cases). Demographically, sex distribution was similar (4 males, 3 females) and no specific comorbidity pattern was observed. Lympholysis with moderate-to-marked splenic white pulp depletion was present in 9 koalas. Endocrine lesions were rare; the most striking being loss of adrenal chromaffin cells in 4.0% (21/519) of cases.

### Fate of koalas

For only 173 of the 519 koalas, the fate of koalas was specified in hospital records as being either dead on arrival (N = 63) or being euthanized (N = 110). However, we were able to identify koala fate for 94.6% (N = 491) of koalas based on clinical history specified in the hospital records (N = 351) or deduced at necropsy cases (N = 140) based on the severity of lesions observed for natural death (i.e. massive skull fracture), or by observing barbiturate crystals within tissues for euthanasia. Confounding factors (e.g. evisceration, previous necropsies) hampered the ability to determine koala fate in 28 cases. A total of 80% (393/491) koalas were euthanized, compared to 20.0% (98/491) being dead on arrival or dying during hospitalization. Of the koalas diagnosed with infectious disease (versus all other conditions), 75.6% (297/393) were euthanized and 26.5% (26/98) were dead on arrival (p < 0.001), while for koalas with trauma (versus all other conditions), only 27.0% (106/393) were euthanized, but 80.6% (79/98) were already dead on arrival or died during hospitalization.

## Discussion

In the current study, the use of systematic necropsies in passive surveillance increased the sensitivity of disease detection and accurately determined comorbidity patterns compared to a previous analysis using medical records where only brief notes on disease were recorded in some animals. Further, systematic necropsies allowed the characterization of several novel diseases and lower frequency etiologies of infectious, neoplastic or idiopathic disease^10,11,15,16^.

Pathology data was recorded using a previously developed rapid classification scheme that streamlined data analysis^11^. The combination of a standardized data recording methodology with systematic necropsies, alongside medical record analysis and ancillary testing, permitted a high (>90%) proportion of conclusive necropsy diagnoses in this study, an excellent rate for wildlife mortality investigations. Other free-ranging wildlife necropsy studies report 46% to 95% rates of determination of primary cause of death^10,17–20^.

The present study interrogated the interplay of concomitant disease conditions in SEQLD koalas, demonstrating a high rate of comorbidities and reinforcing the complexity and variability of threats to the species. As a single entity trauma was the most common diagnosis in the study, followed by infectious disease. Comorbidities were primarily due to multicentric infections, debilitation, co-occurring trauma and infections. Comorbidities were more common in older animals but still surprisingly prevalent in young koalas, where nearly half had more than one presenting problem. At a population level these conditions would limit breeding potential and increase vulnerability to disease or predation^21^.

Concomitant conditions in koalas have been investigated previously^21–23^. A higher proportion of Queensland koalas with loss of BC from comorbidities such as chronic infections or neoplastic disease has been reported than southern states^10,13,24^. Direct comparison of comorbidity rates with previous studies is complicated due to the different study designs used. Nonetheless, the determination of variation in comorbidity categories between studies and over time assists in establishing a historical pattern for disease interaction in koalas^10^. Reports from northern koala populations using smaller sample sizes have found comorbidity proportions ranging from 38–60% in QLD^13,24^ and 19–53% in NSW^21,22,25^. The proportion of comorbidities found in this study (58%) and our former retrospective epidemiological analysis (38%) is within the documented QLD range. Given the large cohort of koalas assessed and the detailed assessment in the present study, higher accuracy is likely in this analysis.

Historically, trauma has been a major contributor to koala mortality in SEQLD^10,11^. When koalas inhabit forested land in close proximity to urbanized centers, there is high mortality from trauma, particularly by motor vehicles and dog predation, demonstrating poor adaptability as these threats permeate into their habitat^10,26^. Koala-motor vehicle collisions have a high fatality rate (>80%)^10,27^ from a combination of factors^22,28^, including comminuted fractures^21,22^ and severe soft tissue trauma causing massive hemorrhage and shock. Dog predation also causes severe soft tissue damage, often leaving pathognomonic bite wound lesions^29^. Although most animal attacks on koalas in urban areas come from domestic dogs^30–32^, predation threat could also permeate into protected forests or bushland habitat when carried out by wild dogs and dingoes^21,33^. Koalas are crepuscular species^1^ thus responsible pet ownership that restricts dog movements on private land during dawn and dusk may be beneficial^10^. At younger ages, both male and female koalas are at risk of trauma during post-weaning dispersal^30^. However, the predominance of mature male koalas in the trauma group could reflect larger home ranges and increased mobility during breeding season^34,35^. Younger males also tend to disperse more frequently and further than female koalas^36^. The removal of subadult males by trauma could disrupt population genetic heterogeneity, as these koalas are the primary inter-group dispersers contributing to gene flow^36^. Notably, in both the medical record and necropsy studies, over half of the animals killed by motor vehicle trauma were healthy. The proportion of healthy animals was also high in animal attack and other trauma causes. Thus, many trauma cases were unnaturally removed from the population predominantly due to anthropogenic factors, potentially causing detrimental impacts on the survival of local populations by reducing healthy breeding stock.

The most frequently reported infectious disease in koalas is chlamydiosis caused by *Chlamydia pneumoniae* producing conjunctivitis and pneumonia, or the more pathogenic *C. pecorum*, which causes conjunctivitis, cystitis and reproductive infection resulting in infertility^37–39^. Classical presentations of clinical chlamydiosis in this study were typically chronic and severe. This reflects the bias of sick wild koalas coming to attention of the public and wildlife rescuers, as well as the clinical management of severely debilitated koalas by euthanasia. In this study morphologic confirmation of typical chlamydial lesions was considered sufficient for diagnosis. Molecular testing for chlamydiosis is of limited value in our context of interrogating drivers of mortality, as subclinical carriers are common and PCR detection and chlamydial load do not correlate with clinical severity^40,41^. For example, koalas with substantial chronic *Chlamydia*-associated structural pathology may be PCR-negative and lack chlamydial inclusion bodies, and conversely higher loads can be observed in infected asymptomatic animals then those with clinical disease^40–42^. A recent study on *Chlamydia pecorum* in the male koala reproductive system demonstrated 70% of morphologically normal samples and 89% of abnormal samples were qPCR positive^43^. Typically studies utilizing active surveillance document elevated frequencies of milder chlamydial infections, compared with this study emphasizing bias in sampling methodology^44^. This dataset will overestimate the proportion of severe end-stage conditions, and underestimate subclinical carriers, but was appropriate for identifying the impact of chlamydiosis as a cause of mortality and infertility^10^.

A disproportionate number of female koalas of breeding age were diagnosed with chlamydial reproductive disease alone or as a comorbidity^10,44–46^ compared to males, despite the documented similarity in infection rates across sexes^38,47^. This reflects the use of ultrasonography to identify permanent female infertility and clinical management by euthanasia^10,45,46,48^. Additional features influencing female bias in reproductive chlamydiosis include urogenital anatomy, increased rate of opportunistic infections, and lack of detectable sonographic changes in the male unless severely affected^44,49,50^. However, clinical and subclinical male reproductive tract disease was detected with histopathology. The impact of male urogenital chlamydosis on individual and population health warrants further investigation. Specifically diagnostic testing of hospitalized male koalas and evidenced-based evaluation of the value of treatment of asymptomatic infections should be explored^11,44,48^.

Cystitis, the main clinical manifestation compatible with chlamydiosis, was found in similar proportion (34%) to other reports^21,23,51^ but higher compared to that described in an previous study by the authors (27%)^10,11^. This difference is likely due to the increased sensitivity of necropsies. Notably, a higher proportion of males had ocular and urinary infections alone or as comorbidities. Previous QLD reports questioned the relationship between low BC and ophthalmic chlamydiosis^52^. In the present study, only 9% of individuals with solely ocular disease had low BC, whilst >60% of koalas with low BC had chronic ocular chlamydiosis associated with comorbidities. Thus, general systemic disease rather than visual impairment causes low BC compromising survivorship^52^. This study has reinforced that chlamydiosis is still the major impactor on koala mortality and morbidity, with a high rate of multicentric disease and comorbidity.

Koalas live on a highly specialized, nutrient-restricted diet, and have limited visceral fat reserves^10,52^. As a result, koalas are prone to debilitating and sometimes irreversible BC loss, known as wasting^10,11,53–55^. Factors causing loss of BC include stressors (prolonged veterinary treatment), or those derived from biotic (social dynamics, resource availability, predation), climatic factors, and dental attrition reducing digestion efficiency^56–59^. Thus, BC is a relevant parameter to assess in koalas^10,11,60^ as it may impact successful rehabilitation, population management, or even interact with comorbid states reflecting disease severity. In this study a strong correlation between the presence of disease, comorbidities, type of trauma and body condition was present. Virtually half of diseased koalas (48%) in the present study had poor BC, and animals with poor BC were more likely to have died from non-traumatic causes. In contrast, 95% of the healthy koalas with trauma had a fair to excellent BC. A larger proportion of koalas with low BC were involved in animal attacks, tree fall and other trauma, confirming an elevated risk with increased ground time due to debilitation in increasingly urbanized landscapes^21^. Conversely, vehicle collisions generally affected animals in good or excellent BC, suggesting these may be active and robust animals moving in search of mates or forage. Notably, male koalas in the young/subadult age class were diagnosed most frequently with idiopathic low BC, exhibiting non-specific clinical histories and lacking necropsy findings. Previous studies reported idiopathic low BC primarily in females, and associated with lymphoid depletion, though this was not consistent in the young males in this study^21,23,24,44^.

Passive surveillance methodology has limitations including minimal control over data quality, lack of denominator values to estimate disease frequency, and overrepresentation of certain demographic groups or diseases (i.e. incurable, terminal)^10,59^. For instance, chronic disease and low BC were observed at high frequency, reflecting the biased selection of terminal animals. Conversely, acute infectious disease was underrepresented, reflecting a bias against individuals which could be successfully rehabilitated^52^. Carcass preservation is often a challenge with wildlife sampling. In koalas, the quality of tissues available for study may have sporadically compromised diagnostic sensitivity due to poor preservation, freezing artefact or eviscerated from previous necropsies, or pap collection for orphaned koalas. In spite of these limitations, utilizing hospital admissions and standardized nomenclature permitted the acquisition of valuable data at the population level in a cost-effective manner, with a high diagnostic success rate. Necropsies proved to be an effective method for the detection of subclinical conditions and identification of comorbidities. In conclusion, management of threats such as trauma, disease, and rapid clearing of koala habitat, need to be tackled in an integrated koala conservation strategy^5^. This study has quantified major causes of mortality and morbidity and the interaction of comorbidities, reflecting the complex threats acting cumulatively against the SEQLD koala population^5–7,61,62^.

## Materials and Methods

### Survey population

Cases for the necropsy survey were sent to the School of Veterinary Science, The University of Queensland, Gatton, from three wildlife hospitals (Moggill Koala Rehabilitation Centre, Currumbin Wildlife Sanctuary Hospital, and Australia Zoo Wildlife Hospital) in Queensland, Australia. Koala admissions to these hospitals derived mainly from SEQLD, but also included some regions in NSW in close proximity to the QLD border. Koalas assessed in this study were either found dead, died during hospitalization, or were euthanized. All samples were collected under a Scientific Purposes Permit from the Department of Environment and Heritage Protection, Queensland Government (WISP 13247813, generated 07/08/2013), now known as the Department of Environment and Science, and under ethical review of The University of Queensland Animal Welfare Unit (ANRFA/SVS/193/13/EHP).

### Necropsies and sample collection

Necropsies were conducted at the School of Veterinary Science, The University of Queensland, between April 2013 and July 2016. The majority of carcasses were frozen at −20 °C due to logistical reasons, and thawed between 0–4 °C for 4–7 days prior to examination. Koalas were identified by ear tags, microchip numbers, or by the hospital accession numbers. Aging was done according to premolar and molar tooth wear designating koalas to an age class (young/subadult, mature, and old)^10,56,62^. Body condition (BC) score was recorded from 1 through 10 by palpating the infraspinatus and supraspinatus muscles and ranged from emaciated 1–2, poor 3–4, fair 5–6, good 7–8 and excellent >8 BC^60,63^.

Histopathology samples were fixed in 10% NBF, sectioned at 4 µm and stained with hematoxylin and eosin by routine methods. Special stains performed included: Periodic Acid Schiff’s (PAS), for mucins, mucopolysaccarides and fungal agents, Gram’s for infectious agents, Masson’s Trichrome for collagen and smooth muscle, and Ziehl-Neelsen for acid fast organisms. Ancillary testing included bacterial and fungal culture by routine methods at Veterinary Laboratory Services, The University of Queensland (Gatton, Australia). PCR (panfungal, bacterial) assays were conducted at Pathology West (Sydney, Australia) for the molecular identification of bacteria and fungi. Panfungal PCR targeted fragments of the rDNA gene cluster, specifically, those internally transcribed in spacer region located between the 18S and 28S^10,64^. The bacterial PCR assay targeted conserved regions U1 and U3 for the 16S rRNA gene using broad range primers^10,65^. Variable portions within the 16S rRNA gene provide unique signatures of any bacterium. Identification was conducted via sequence analysis of the amplified product^10,65^.

### Clinical disease grading criteria

Chlamydial conjunctivitis and cystitis was subjectively scored by hospitals based on the degree of tissue damage and disease progression following the disease grading criteria of the Moggill Koala Rehabilitation Centre Standard Operating Procedures for conjunctivitis and cystitis treatment 2017 (Supplementary Table 2) as well as previous reports^44^. Chlamydial bursitis was not clinically-scored as it causes permanent infertility regardless of the degree, with affected females being unable to be returned to the wild, most being euthanased with barbiturates, as per the discretion of the consulting veterinary surgeon under the Veterinary Surgeons Act of Queensland.

### Data analysis

Binomial, or also called exact or Clopper-Pearson confidence intervals were calculated for the overall prevalence of observed co-morbidities and for co-morbidities stratified by sex and age^66^. Exact confidence intervals are more appropriate for small proportions (as for the co-morbidity strata in our study), but as they more conservative, they might be wider than traditional Wald-type intervals^67^.

Analyses of relationships between the various diagnoses made and demographic information of koalas submitted (sex, age, age class, body condition) were conducted using contingency tables. Fisher exact tests were used to compare proportions in the contingency tables^68^. Data analysis was conducted in STATA 15.0 (StataCorp. 2017. Stata Statistical Software: Release 15. College Station, TX: StataCorp LLC).

## Supplementary information

Supplementary Material

## Data Availability

The authors agree to comply with the publication’s requirements for sharing materials.
