# Supplementary material for: A Necropsy Study of Disease and Comorbidity Trends in Morbidity and Mortality in the Koala (Phascolarctos cinereus) in South-East Queensland, Australia
Source: Sci Rep. 2019 Nov 25;9:17494. doi: 10.1038/s41598-019-53970-0 (PMC6877607; doi:10.1038/s41598-019-53970-0)

A Necropsy Study of Disease and Comorbidity Trends in Morbidity and Mortality in the Koala (*Phascolarctos cinereus*) in South-East Queensland, Australia.

V. Gonzalez-Astudillo,J. Henning, L. Valenza, L. Knott, A. McKinnon, R. Larkin, R. Allavena

Supplementary Table S1: Single (Total: N=220) and co-ocurring (Total: N=299) diagnoses made of koalas submitted to wildlife hospitals and receiving necropsies at The University of Queensland between April 2013 and July 2016 in South-East Queensland, Australia.

Diagnoses	Co-morbidity (N diagnoses)	
	Absent	Present
Infectious	31.8% (70)	
Infectious (multicentric)		32.1% (96)
Infectious/neoplasia		4.3% (13)
Infectious/neoplasia/senescence		1.0% (3)
Infectious/neoplasia/senescence/other diseases		0.3% (1)
Infectious/other diseases		7.7% (23)
Infectious/senescence		15.1% (45)
Infectious/senescence/other diseases		3.0% (9)
Infectious/senescence/trauma		1.3% (4)
Infectious/trauma		18.4% (55)
Infectious/trauma/other diseases		2.0% (6)
Infectious/trauma/senescence/other diseases		0.7% (2)
Neoplasia	6.4% (14)	
Neoplasia/infectious		0.3% (1)
Neoplasia/infectious/other diseases		0.3% (1)
Neoplasia/other diseases		0.7% (2)
Neoplasia/senescence		1.3% (4)
Neoplasia/trauma		0.7% (2)
Other diseases	4.1% (9)	
Senescence	3.6% (8)	
Senescence/other diseases		1.7% (5)
Trauma	42.7% (94)	
Trauma/other diseases		3.0% (9)
Trauma/senescence		1.0% (3)
Undetermined	3.6% (8)	
Wasting unknown cause	7.7% (17)	
Wasting unknown cause/other diseases		0.3% (1)
Wasting unknown cause/trauma		4.3% (13)
Wasting unknown cause/trauma/other diseases		0.3% (1)

Supplemental Material S2. Clinical guidelines for the diagnoses of conjunctivitis and cystitis in koalas according to the Standard Operating Procedures by the Moggill Koala Rehabilitation Center, in Queensland, Australia, 2017.

Condition	Score	Severity	Criterion
Conjunctivitis	1	Mild	Acute membrana nictans inflammation lacking proliferative change with epiphora
	2	Moderate	Mild conjunctiva and membrana nictans proliferation with serous exudate,<br>Keratitis may or may not be present, or periorbital alopecia
	3	Severe	Chronic conjunctiva and membrana nictans inflammation<br>Mucopurulent discharge causing eyelid adhesion,<br>Possible conjunctival bleeding,<br>Keratitis leading to partial or total corneal opacity,<br>Focally extensive periorbital alopecia
Cystitis	1	Mild	Common vestibule is slightly or moderately soiled
	2	Moderate	Soiled common vestibule, incontinence evident from dribbling micturition, urine odor present, tacky fur
	3	Severe	Soiled common vestibule with matted fur, fur loss,<br>Excoriation, incontinence, hemorrhagic dysuria, associated with low BC

Supplementary figure S1: Percentage of koalas with gross lesions compatible with chlamydiosis across all body systems affected, stratified by sex. Koalas were submitted to South-East Queensland hospitals and receiving necropsies at The University of Queensland from 2013 through to 2016 (N=304).

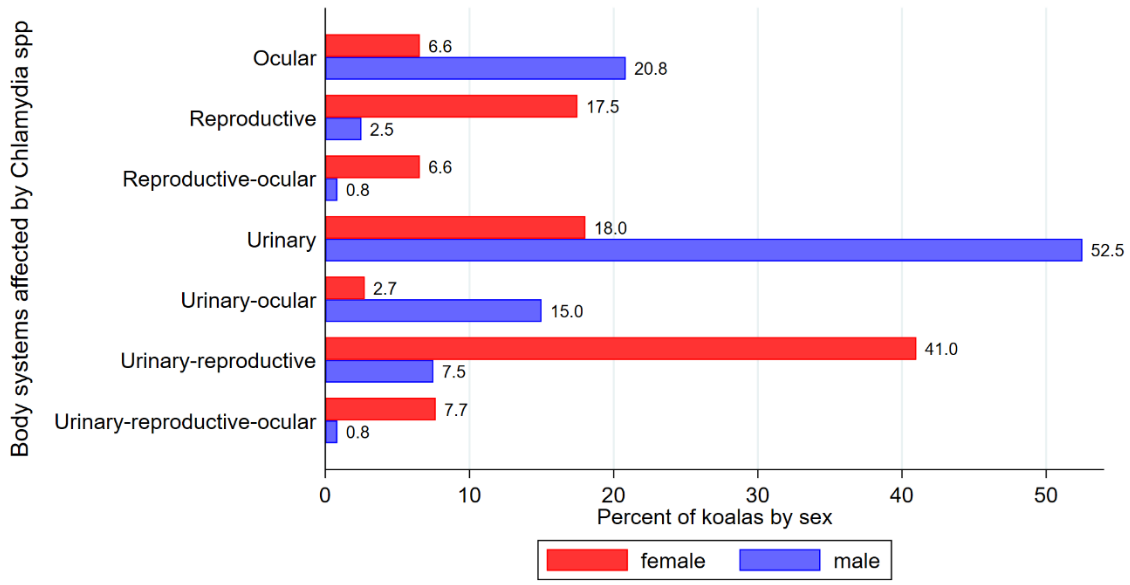

Supplement: Supplementary file 1 — Supplementary Material [file 41598_2019_53970_MOESM1_ESM.pdf]
